# Among the Colleges

**Published:** 1902-10

**Authors:** 


### AMONG THE COLLEGES.

University Veterinary College, Kansas City, Missouri. First annual announcement received—Session 1902–03. G. W. Werner, President; J. V. Cowles, Vice-President; W. J. Wilson, Secretary; W. J. Gatchell, Treasurer.

Western Veterinary College, Kansas City, Mo. Sixth annual announcement received. Changes in faculty—Albert A. Immel, D.V.S., Bacteriology, in place of James B. Black, V.S.; Theodore W. Hadley, Veterinary Materia Medica, in place of Frank D. Rowell, D.V.S., M.D. Added, Joseph S. Snider, M.D., Histology, and Wm. P. Cutler, M.D., Hygiene and Sanitary Science. Dropped, James B. Black, V.S., Operative Surgery.

The Western Veterinary College opens its Fall Session of 1902 in its new building in Kansas City, Mo. The building has been specially constructed for the maintenance of the school.

Chicago Veterinary College, Chicago, Illinois. Catalogue session 1902–03 received. No special changes are noted. Post-graduate course will be continued in connection with the regular course.

Ontario Veterinary College, Toronto, Canada. Catalogue session 1902–03 received.

Collins Veterinary Medical College, Nashville, Tennessee. Fourth annual announcement received.

Veterinary Department, University of Pennsylvania, Philadelphia, Pa. Announcement for the eighteenth annual session, 1902–03, received. No special changes noted.

